# Data Fusion of Two Hyperspectral Imaging Systems with Complementary Spectral Sensing Ranges for Blueberry Bruising Detection

**DOI:** 10.3390/s18124463

**Published:** 2018-12-17

**Authors:** Shuxiang Fan, Changying Li, Wenqian Huang, Liping Chen

**Affiliations:** 1 Beijing Research Center of Intelligent Equipment for Agriculture, Beijing 100097, China; fanshuxiang@outlook.com (S.F.); huangwq@nercita.org.cn (W.H.); chenlp@nercita.org.cn (L.C.); 2 Bio-Sensing and Instrumentation Laboratory, College of Engineering, University of Georgia, Athens, GA 30602, USA

**Keywords:** blueberry, bruising, data fusion, hyperspectral imaging

## Abstract

Currently, the detection of blueberry internal bruising focuses mostly on single hyperspectral imaging (HSI) systems. Attempts to fuse different HSI systems with complementary spectral ranges are still lacking. A push broom based HSI system and a liquid crystal tunable filter (LCTF) based HSI system with different sensing ranges and detectors were investigated to jointly detect blueberry internal bruising in the lab. The mean reflectance spectrum of each berry sample was extracted from the data obtained by two HSI systems respectively. The spectral data from the two spectroscopic techniques were analyzed separately using feature selection method, partial least squares-discriminant analysis (PLS-DA), and support vector machine (SVM), and then fused with three data fusion strategies at the data level, feature level, and decision level. The three data fusion strategies achieved better classification results than using each HSI system alone. The decision level fusion integrating classification results from the two instruments with selected relevant features achieved more promising results, suggesting that the two HSI systems with complementary spectral ranges, combined with feature selection and data fusion strategies, could be used synergistically to improve blueberry internal bruising detection. This study was the first step in demonstrating the feasibility of the fusion of two HSI systems with complementary spectral ranges for detecting blueberry bruising, which could lead to a multispectral imaging system with a few selected wavelengths and an appropriate detector for bruising detection on the packing line.

## 1. Introduction

The blueberry is an important horticultural crop in the United States of America. Its production was 262,539 tons in 2014, accounting for about 50% of the world production [1]. Mechanical systems have been widely used in blueberry harvesting to reduce labor cost, but low harvest efficiency and high incidence of fruit bruising contributes to the majority (78%) of bruising in mechanically harvested blueberries, affecting the overall quality and shelf life of harvested blueberries [2]. Additionally, blueberries are prone to impact and mechanical damage during transportation when the fruit collides with hard surfaces. Therefore, in order to remain competitive, careful postharvest handling is required and bruised fruit should be sorted out before they are sold in the fresh market.

Because the skin of the blueberry is dark and opaque to most visible light, internal bruises under skin are not visible to the human eyes. Thus, it is challenging for traditional RGB images to detect blueberry bruises nondestructively [3]. Over the past decades, hyperspectral imaging (HSI) that integrates imaging and spectral technologies has become a powerful tool for quality inspection of fruits and vegetables [4]. Push broom based HSI, also called line scan configuration, acquires the spectra along a sample line at a time and forms a special 2-D image with one dimension in space and one dimension in spectrum. A complete hyperspectral cube can be obtained as the imaging system scans the entire sample line by line along the moving direction of the tested sample, making it particularly suitable for conveyor belt systems. Therefore, push broom based HSI, usually with a spectral range of 400–1000 nm, has been predominantly used for bruise detection in apples and pears, and defect inspection of oranges and peaches [5].

On the other hand, electronically tunable filter (ETF) based technology is another configuration of hyperspectral image acquisition, which involves keeping the image field of view fixed, and obtaining images with 2-D spatial information at a single wavelength at a time. The ETF-based HSI systems can select spectral bands randomly as well as continuously. As the detector is exposed to only a single wavelength each time, a suitable exposure time can be set for each wavelength. Therefore, they are more compact and have higher potential for field applications [6]. As the liquid crystal tunable filter (LCTF) system has larger apertures, relatively wider field of view, and lower wavefront distortions, it has become the most commonly used ETF and has been successfully used to detect bruises in strawberries [7], bacterial diseases in onions [8], and decay in mandarins [9]. 

Both push broom based and LCTF based hyperspectral imaging techniques have been applied to blueberry bruise detection. Hu et al. [10] compared reflectance and transmittance as well as interactance push broom based hyperspectral imaging over the wavelength region of 680–1100 nm in characterizing and detecting non-visible mechanical damage of blueberries with time evolution. It was found that transmittance-based classifiers obtained satisfactory accuracies for classifying damaged and sound blueberries 2 days after impact.

Previous work conducted in our lab has demonstrated that LCTF based hyperspectral reflectance imaging system in the spectral range of 950–1650 nm could be implemented to detect blueberry bruises 24 h after the impact [11]. The predicted bruise ratio index had a strong correlation with human assessment (R^2^ = 0.78–0.83, root mean square error (RMSE) = 0.104–0.125). The successful application of LCTF based hyperspectral transmittance mode was also achieved in our lab to detect stem and equator bruises by thresholding the images at 1070 nm with R^2^ = 0.819, RMSE = 0.116 for bruise ratio index prediction [12]. The aforementioned literature demonstrated that both spectral ranges of 680–1100 nm and 950–1650 nm could be used to detect the blueberry bruising. However, to the best of our knowledge, no research has been reported yet for blueberry internal bruising detection by fusing different HSI systems with complementary spectral sensing ranges. 

Data fusion is a process of combining information from different individual sources to provide a robust and complete description of an environment or process of interest, rather than using an individual source alone [13]. Because diverse sources of data usually provide more detailed and potentially complementary information compared with using a single analytical technique [14], data fusion has been applied in civilian surveillance and monitoring tasks, in process control, and in information systems [15,16]. Although the concept of data fusion was proposed decades ago, the technology is still in its infancy in terms of increasing the reliability of classification or prediction of foodstuff specifications, and is gaining popularity towards fast and non-destructive evaluation of fruit quality [17,18]. By using a majority voting process to combine the classifiers associated with three different kinds of firmness sensors (based on sound, impact, and micro-deformation), the classification error rate for firmness of peach dropped from around 20% when a single sensor was used, to 14% when the three sensors were fused [19]. The Electronic nose (Enose) and another type of chemical sensor called zNose^TM^ data were fused to improve detection and classification performance for damaged apples by using multi-sensor data fusion models at feature level and decision level. The results showed that the feature level fusion with the covariance matrix adaptation evolutionary strategy and decision level fusion using a Bayesian network as a classifier reduced the classification error rate by 13% and 2%, respectively [20]. A Bayesian statistical approach was implemented to incorporate two sensors, colorimeter and acoustic impact, to detect the maturation stages of tomatoes; the classification error was decreased from 48% to 11% after data fusion [21]. Four nondestructive technologies (acoustic firmness, bioyield firmness, Vis-SWNIR spectroscopy, and spectral scattering) were fused by simply concatenating the raw signals provided by different sensors after pre-processing to improve the soluble solids content and firmness prediction of apples, with the standard error of prediction (SEP) reduced by 6.2–24.9% and 5.8–6.0%, respectively [18]. The aforementioned studies showed that multiple sensors could provide more knowledge about a sample, substantially enhancing the reliability of classification or prediction results by overcoming the limitations of a single technique. In addition, there has been very little research directly exploring the fusion of hyperspectral images with different spatial and spectral resolutions in nondestructive sensing of food quality, lacking effective data fusion approaches to fuse hyperspectral data sets.

Currently, a typical hyperspectral imaging system rarely covers the visible and near-infrared regions from 400 to 2500 nm, due to the challenge of making a sensor that is sensitive to this wide spectral range. As a wide spectral range would usually provide more comprehensive and useful information of tested samples, it makes sense to use two detectors that are sensitive to wavelength ranges of 400–1000 nm and 900–2500 nm, respectively. With the help of data fusion algorithms, the acquired spectral data with different wavelength ranges from the push broom based and LCTF based HSI systems could be fused in data, feature, and decision levels to provide complementary information for developing a more accurate and robust classification model for blueberry bruising detection.

The long acquisition time required to collect high-dimensional hyperspectral images poses considerable challenges to real-time inspections, limiting the hyperspectral imaging technology to commercialized packing lines that require a fast speed. As a result, hyperspectral imaging is always used for off-line applications and optimal wavelength selection is usually performed to form multispectral images. A multispectral imaging system aims to acquire spatial and spectral information that is directly useful for a specific application. It only captures images at the selected wavelengths (usually under ten discrete wavelengths), thus reducing the total volume of the data and improving the processing speed and efficiency. The multispectral imaging system usually consists of appropriate detectors that are sensitive to only a few effective wavelengths selected from the HSI system and the detectors could be Si-based or InGaAs-based charge-coupled device (CCD) cameras. Because of the low cost and fast computing speed in comparison with the HSI system, several prototypes of multispectral imaging systems have been developed to detect citrus canker [22] and apple defects [23,24], and to predict apple fruit firmness [25]. Meanwhile, commercial multispectral cameras have become more customizable and flexible, and could support the configuration of customized user-selected filters for specific applications. Therefore, the effective features should be determined from the HSI systems before developing a multispectral imaging system to detect blueberry bruising on the packing line.

The overall goal of this research was to evaluate the efficacy of data fusion strategies for the HSI systems to improve blueberry bruising detection. The specific objectives were to: (1) obtain the mean spectrum of each blueberry from push broom and LCTF based hyperspectral data respectively, (2) select and determine effective features from the two extracted spectral data sets with complementary spectral ranges, and (3) compare the performance of three data fusion strategies at the data level, feature level, and decision level, by combining the two spectral data sets.

## 2. Materials and Methods

### 2.1. Samples

Two varieties of blueberries, Bluecrop and Jersey, harvested in Michigan, USA, in August 2016 were packed in clamshells and shipped to the Bio-Sensing and Instrumentation Lab at the University of Georgia. All of the blueberries that were used in the experiment were taken out of the refrigerator and stored in the air-conditioned laboratory for 2 h before treatment (bruise or control, as described in the following paragraph) to allow the samples to reach room temperature (20 °C). 

**Figure 1:**
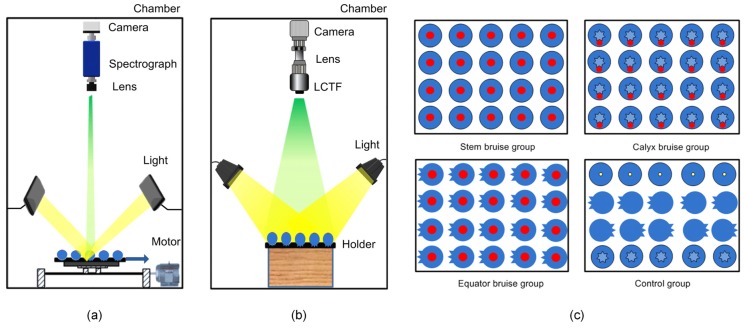
Schematic diagram of the (**a**) push broom based and (**b**) liquid crystal tunable filter (LCTF) based hyperspectral imaging system, and (**c**) blueberry orientations used for acquiring images.

A total of 320 Bluecrop blueberries and 384 Jersey blueberries were used in the experiment. Each variety was divided equally into four groups and each group was assigned to one of four time treatments. Therefore, each time treatment group contained 80 Bluecrop and 96 Jersey blueberries. For each of the four time treatment groups, the 80 Bluecrop and 96 Jersey blueberries were divided equally into four groups and each group was assigned to one of four physical treatments: stem bruise, equator bruise, calyx bruise, and control (no bruise treatment, also referred to as ‘healthy’). Therefore, each physical treatment group contained 20 Bluecrop and 24 Jersey blueberries. A steel ball, mounted on a wooden pendulum, was designed to create bruising by dropping the steel ball from a height of 90 mm onto a specific position (according to physical treatment group) of each blueberry’s surface. A holder, made of the silicon rubber, was put under the tested sample to avoid damage at the bottom of the sample when the bruise was created. The blueberry samples were stored in the laboratory for 30 min, 2 h, 6 h, or 12 h (according to time treatment group) after bruise creation, before they were imaged with both push broom based and LCTF based HSI systems. Samples from the same physical treatment group were placed on one specially designed black cardboard holder with the bruised surfaces facing the camera. It should be noted that it is difficult to create bruises at the calyx end due to the existence of vestigial sepals around the calyx. Thus, the bruises were created under the sepals for calyx bruise groups in this study (Figure 1c). For the control group, four fruit orientations (stem, calyx, equator, and back equator facing the camera) were taken into consideration (Figure 1c). In order to balance the number of bruised and healthy samples, 60 bruised and 60 control samples were selected randomly from each variety as the calibration set to develop classification models, and the remaining samples were used as the prediction set to validate the model (see Supplementary Table S1). 

### 2.2. Hyperspectral Image Acquisition

#### 2.2.1. Push Broom Based HSI

A push broom based HSI imaging system (Figure 1a) has been developed in our lab [26]. The system was composed of a 14-bit CCD camera with 1392 × 1040 pixel resolution (ICL-B1410, Imperx Inc., Boca Raton, FL, USA), a prism-grating-prism imaging spectrograph (ImSpector V10E, Specim, Oulu, Finland) which had a spectral range of 400–1000 nm with a nominal spectral resolution of 2.8 nm, a 150 W quartz tungsten-halogen light source (Fiber-Lite DC950, Dolan-Jenner Industries, Boxborough, MA, USA) illuminating the samples through a bifurcated line light guide, and a sample holder which is integrated with a stepper motor (MDrive 23 Plus, Schneider Electric Motion, Marlborough, CT, USA) and a liner slider (MS33, Thomson Industries Inc., Wood Dale, IL, USA) to move samples to scan one line at a time. The whole HSI system was fixed in a chamber to block ambient light. Custom software was developed using LabVIEW (National Instruments Inc., Austin, TX, USA) for acquiring spectral image acquisition, and controlling camera and motor.

Hyperspectral images were first calibrated by using a white and dark reference to remove artifacts caused by non-uniform illumination or variations in the pixel-to-pixel sensitivity of the detector. For each hyperspectral imaging system, prior to collecting images, the white reference image (*R_w_*) was acquired from a Teflon white panel (SRT-99-050, Labsphere Inc., North Sutton, NH, USA) with a reflectance of 99%, and the dark reference image (*R_d_*) was obtained with the lamps turned off and the optical lens completely covered by its cap. The hyperspectral images (*R_raw_*) were captured by manually placing the black cardboard holder in the liner slider, which moved with a speed of 2.67 mm/s, making a 3D hyperspectral cube (*R_raw_*), which was constructed with spatial dimensions of 1392 × 1040 pixels and spectral dimensions of 256 wavelengths. Then the corrected image (*R_c_*) was calculated according to the following equation:

(1) $$R_{c}=\frac{R_{raw}-R_{d}}{R_{w}-R_{d}}$$

#### 2.2.2. LCTF Based HSI

A short-wave near-infrared LCTF based hyperspectral imaging system has been set up in our laboratory and employed for acquiring hyperspectral reflectance images of blueberries (Figure 1b) [11]. In this study, we used the same system to acquire hyperspectral images of Bluecrop and Jersey blueberries in wavelengths of 965–1650 nm with a 5 nm spectral resolution. The size of each hyperspectral cube was 320 × 256 × 139 data points. The LCTF hyperspectral images were corrected using the white and dark references as described earlier.

### 2.3. Processing of Hyperspectral Images

It was necessary to remove the image background before further analysis. Single-band grayscale images at 820 nm and 1075 nm were used to create mask templates for both push broom based and LCTF based hyperspectral images, due to the prominent contrast between the fruit and the background in these two images. After erosion by removing small objects from the images, the processed images were used as masks to segment individual samples from the background. The acquisition of mask images and segmentation was processed in MATLAB 2016b (The MathWorks Inc., Natick, MA, USA). Subsequently, the spectrum of each pixel within a sample was extracted and then averaged as the mean reflectance for the sample. As the signal to noise ratio was rather low at the beginning and end of the waveband for push broom based HSI, only the 480–960 nm region was considered. Given that the spectral profile of the calyx end collected from LCTF hyperspectral images was similar to that of bruised tissues, the calyx end was always misclassified as bruised tissue and needed to be excluded in image classification. After some trial and comparison, the two-band ratio between 1200 nm and 1075 nm (1200 nm/1075 nm) was used to identify the sepal positions, which surrounds the calyx end.

### 2.4. Reference Measurements

About 1 h after being scanned by the two HSI systems, blueberries were cut in half perpendicular to the bruise position to obtain the ground truth of the samples. Then the sliced samples were imaged with a digital camera (D40, Nikon, Tokyo, Japan). The number of pixels of the discolored (bruised) area and the number of pixels of the entire cross-section of the sliced blueberry were obtained by using ROI function in Environment for Visualizing Images software (ENVI 4.7, Exelis Inc., Tysons Corner, VA, USA). The ratio between the two numbers of pixels was used as the measured bruise ratio of the tested sample. Furthermore, the samples were classified as healthy or bruised according to whether the discolored area accounted for less than (healthy) or greater than (bruised) 25% of the pixels of the sliced surface [27].

### 2.5. Classification Methods

#### 2.5.1. PLS-DA

Partial least squares discriminant analysis (PLS-DA)—an extension form of PLS modeling which aims to find the variables and directions in a multivariate space that discriminate the known classes—has been widely used in the quality control of food commodities. In PLS-DA, a PLS regression model is calculated that relates the independent variables (e.g., spectra) to an indicator Y matrix of category variables encoded as +1 and −1. In this study, the number +1 is used to indicate that the training sample belongs to the healthy class, and the number -1 indicates that the sample belongs to the bruised class.

Classification of an unknown sample is derived from the value predicted by the developed PLS model, *y*. This value is a real number, not an integer, which should ideally be close to the values used to codify the class. In the present study, an advanced approach was used to calculate prediction probability and cut-off value [28]. For simplicity, the algorithm assumes that the predictions obtained by the PLS model for each class in the training set follow a Gaussian distribution. Then, the mean and the standard deviation of these predictions are used to estimate a probability density function (PDF) for each class as the probability of observing a value of *y* given a sample from healthy (*h*) or bruised (*b*) class (see Supplementary Figure S1):

(2) $$p\left(y|h\right)=\frac{1}{\sigma_{h}\sqrt{2\pi }}\exp\left(-\frac{{\left(y-\mu_{h}\right)}^{2}}{2\sigma_{h}^{2}}\right)$$

(3) $$p\left(y|b\right)=\frac{1}{\sigma_{b}\sqrt{2\pi }}\exp\left(-\frac{{\left(y-\mu_{b}\right)}^{2}}{2\sigma_{b}^{2}}\right)$$

where *σ_h_*, *σ_b_* and *μ_h_*, *μ_b_* are the standard deviations and means of predictions in the training set obtained by the PLS model for healthy and bruised classes, respectively. According to Bayes theory, the probability that a sample is from healthy or bruised samples given a particular value of *y* is:

(4) $$p\left(h|y\right)=\frac{p\left(y|h\right)p\left(y\right)}{p\left(y\right)}=\frac{p\left(y|h\right)p\left(h\right)}{p\left(y|h\right)p\left(h\right)+p\left(y|b\right)p\left(b\right)}=\frac{p\left(y|h\right)}{p\left(y|h\right)+p\left(y|b\right)}$$

(5) $$p\left(b|y\right)=\frac{p\left(y|b\right)p\left(y\right)}{p\left(y\right)}=\frac{p\left(y|b\right)p\left(b\right)}{p\left(y|h\right)p\left(h\right)+p\left(y|b\right)p\left(b\right)}=\frac{p\left(y|b\right)}{p\left(y|h\right)+p\left(y|b\right)}$$

where *P*(*h*) and *P*(*b*) are the probabilities that we will observe healthy or bruised samples in the population, they are set to 0.5 here. The two probability density function curves intersect in one point where both *P*(*h*|*y*) and *P*(*b*|*y*) are 0.5. This point is selected as the threshold for the PLS-DA so that a sample is assigned to healthy class if the prediction is larger than the cut-off value, or assigned to bruised class otherwise. Therefore, the classifiers provide information on the “strength” of its belief that a particular sample belongs to a certain class. Cross-validation is the most popular method to optimize the number of latent variables (LVs) of the PLS-DA model and to avoid the problem of over-fitting. The optimal number of LVs to be included in each model was determined by 10-fold cross-validation until the classification error of cross validation reached minimum.

#### 2.5.2. SVM

Support vector machine (SVM) is a powerful machine learning method for classification, regression, and distribution estimation. It was implemented in MATLAB with an open source LIBSVM V 3.22 toolbox. The radial basis function (RBF) was selected as the kernel function used for SVM as it frequently handled the non-linear relationship between the spectra and target class. In order to yield the best performance of the SVM classifier, two parameters needed to be optimized: the penalty (*c*) of the model and the gamma (*g*) of the kernel function. The SVM classifier was trained to output the likelihood that a particular sample belongs to a certain class by setting the probability estimates (*b*) parameter in this study. A grid-search approach was applied to the classification dataset to find the best values of *c* and *g*. According to the maximum classification accuracy of 10-fold cross validation under different combinations of *c* and *g*, the best values of *c* and *g* were determined.

### 2.6. Data Fusion

**Figure 2:**
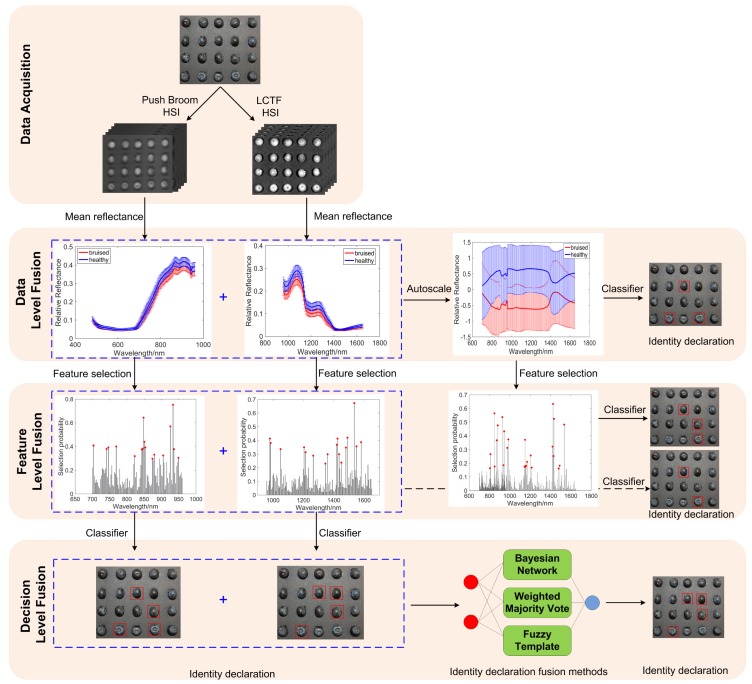
Data fusion processing pipeline.

The fusion of spectral data from the push broom based and LCTF based HSI techniques were performed at these three levels (Figure 2). The three levels of fusion are: data level fusion, feature level fusion, and decision level fusion, also called low-level, mid-level, and high-level fusion [14].

#### 2.6.1. Data Level Fusion

In the data level fusion, at which data integration occurs at the bottom of the data analytical flow, spectra from the two HSI techniques were simply concatenated into a single matrix that has as many rows as samples analyzed and as many columns as independent variables measured by the two instruments. It should be pointed out that before data level fusion, the spectral data from two instruments were auto-scaled to compensate for the scale differences. Afterwards, PLS-DA and SVM were carried out on the resulting matrix to provide the final classification. As the fusion occurs at the level of the original data, the fused matrix will normally contain a very high number of variables. Thus, the main drawback of this strategy is that the increase in information obtained by adding more data to describe the sample may not compensate for the amount of irrelevant variance brought by the addition of the same data [29].

#### 2.6.2. Feature Level Fusion

Selection of important features is of critical significance for removing redundant information from high-dimensional data and optimizing calibration models for improving classification results. In this study, feature selection was conducted to select effective features for blueberry bruise detection using the random frog algorithm, which was developed on the basis of the reversible jump Markov Chain Monte Carlo (RJMCMC) method for selecting cancer-related genes [30]. This method starts with a randomly selected variable subset. Then a new variable subset is generated based on the previous one and is accepted with a certain probability. This step is iteratively continued until predefined iterations are finished. The selection probability for every wavelength was obtained. The advantage of random frog is that no demanding mathematical formulation is needed and no prior distributions need to be specified like in formal RJMCMC methods, making it widely used in spectral variables selection for total soluble solids determination in mulberries [31], firmness detection of blueberries [32], as well as total nitrogen spatial distribution of pepper plants [33]. The detailed explanation of the projection operations is described in the literature [30]. 

In this study, two methods of feature fusion were discussed (Figure 2). For the first method, the features were selected separately from the two HSI systems and then combined together to make a single data matrix with a number of columns equal to the number of features extracted from the two systems. For the other method, the features were extracted jointly from the resulting matrix of data level fusion.

#### 2.6.3. Decision Level Fusion

Decision level fusion in food analysis has mostly focused on classification problems. In decision level fusion, separate classifications are calculated from each data source, and the class outputs from each individual model are combined to obtain the final identity declaration. The classification results can be fused with heuristic techniques. Many studies demonstrated that multiple classifier fusion may generate more accurate classification results [34]. Weighted majority vote, Bayesian network, and fuzzy template strategies were constructed to fuse the outputs of classifiers for cases where the push broom based and LCTF based classifiers were in disagreement. The procedures were developed by the authors to implement these decision level fusion strategies in MATLAB 2016b.

##### Weighted Majority Vote

Majority voting is the simplest form of combining individual classification algorithms, in which choosing the combination rule is critical for designing classifier ensembles. However, it may suffer from higher degradation in noisy environment if all the sensors are weighted identically for all the classes without using previous statistics [35]. Therefore, the weighted majority vote was introduced by considering the prior knowledge of the behavior of the individual classifiers. The combination rule—in which weights are assigned to each classifier and each class—was applied in the present study [36]. In assigning weight values to classifiers, the aim is to assign greater weight values to classifiers with greater predictive performance in terms of an output class. Each classifier is assigned to a weight value in the range of [0, 1] to indicate the classifier’s performance on a particular output class. In that way, weight values for each classifier and class output pairs are kept.

The two classifiers’ estimated performance on a particular output, healthy (*h*) or bruised (*b*) class, were determined by the classification accuracies in cross validation in this study. Let α*^l^*(*i*) means the classification accuracy in cross validation for class *i* using *l*-th classifier. Then, the weights are assigned to each classifier and class

(6) $$w^{\left(l\right)}\left(i\right)=\frac{\alpha^{l}\left(i\right)}{\sum_{l=1}^{2}\alpha^{l}\left(i\right)}\;\;\;i=h,b$$

Once the weights for each classifier decision and class have been computed, the scores of healthy class *S*(*h*, *x*) and bruised class *S*(*b*, *x*) can be calculated for the sample *x*:

(7) $$S\left(h,x\right)=w^{\left(1\right)}\left(h\right)p^{\left(1\right)}\left(h,x\right)+w^{\left(2\right)}\left(h\right)p^{\left(2\right)}\left(h,x\right)$$

(8) $$S\left(b,x\right)=w^{\left(1\right)}\left(b\right)p^{\left(1\right)}\left(b,x\right)+w^{\left(2\right)}\left(b\right)p^{\left(2\right)}\left(b,x\right)$$

where *p*^(*l*)^(*i*, *x*) means the predicted probability that *x* belongs to *i* class by classifier *l*. The final class label is then derived from the class label with the highest score.

##### Bayesian Network

As a result of the structure of the Bayesian network, the conditional probability of occurrence that a blueberry is healthy, given the measured data of push broom based and LCTF based HSI, can be related as follows:

(9) $$P(blueberry_{h}|PB,LCTF)=\frac{P(PB,LCTF|blueberry_{h})P(blueberry_{h})}{P(PB,LCTF)}$$

Similarly, the conditional probability of occurrence that a blueberry is bruised, given the measurement from push broom based and LCTF based HSI, can be related as follows:

(10) $$P(blueberry_{b}|PB,LCTF)=\frac{P(PB,LCTF|blueberry_{b})P(blueberry_{b})}{P(PB,LCTF)}$$

In the first case (blueberry is healthy), because the sensor measurements are assumed independent, we obtain

(11) $$P(blueberry_{h}|PB,LCTF)=\frac{P(PB,LCTF|blueberry_{h})P(blueberry_{h})}{P(PB,LCTF|blueberry_{h})+P(PB,LCTF|blueberry_{b})}$$

The probability of observing the measurements of push broom based and LCTF based HSI given a sample from the healthy class:

(12) $$\begin{array}{ll} P(PB,LCTF|blueberry_{h}) & =P(PB_{h}|blueberry_{h})P(PB\_Classifier_{h})P(LCTF_{h}|blueberry_{h})P(LCTF\_Classifier_{h})\\ & +P(PB_{b}|blueberry_{h})P(PB\_Classifier_{b})P(LCTF_{h}|blueberry_{h})P(LCTF\_Classifier_{h})\\ & +P(PB_{h}|blueberry_{h})P(PB\_Classifier_{h})P(LCTF_{b}|blueberry_{h})P(LCTF\_Classifier_{b})\\ & +P(PB_{b}|blueberry_{h})P(PB\_Classifier_{b})P(LCTF_{b}|blueberry_{h})P(LCTF\_Classifier_{b}) \end{array}$$

The probabilities *P(PB_Classifier_h_) (or P(PB_Classifier_b_))* and *P(LCTF_Classifier_h_) (or P(LCTF_Classifier_b_))* represent the probability outputs that a sample belongs to the healthy (or bruised) class by push broom based and LCTF based hyperspectral data, respectively. On the other hand, the conditional probabilities *P(PB_h/b_|Blueberry_h_)*, *P(LCTF_h/b_|Blueberry_h_)* and *P(PB_h/b_|Blueberry_b_)*, *P(LCTF_h/b_|Blueberry_b_)* incorporate a prior independent probability regarding the performance of the push broom based and LCTF based HSI systems given a healthy and bruised blueberry, respectively. Specificity and sensitivity in the cross validation set were used as these conditional probabilities in this study. The probabilities that we will observe healthy (*P(blueberry_h_)*) or bruised (*P(blueberry_b_)*) samples were set to 0.5 here. For the second case (blueberry is bruised), the derivation is similar to the first case and, therefore, was omitted to avoid redundancy. Ultimately, by comparing the *P(blueberry_h_ |PB, LCTF)* and *P(blueberry_b_ | PB, LCTF)*, the final decision was determined by choosing the higher probability output of the two.

##### Fuzzy Template

Fuzzy template technique is a very simple classifier fusion method that combines the outputs of multiple classifiers [37]. It is likely that through its flexibility and simplicity, fuzzy template method may outperform other more complex fusion methods requiring substantially larger numbers of parameters (e.g., fuzzy integrals) [38]. Let *C* = {*C_1_, …, C_L_*} be the set of L classifiers. The probability output of the *m*-th classifier is *C_m_(x)* = [*d_m,1_(x),…, d_m,c_(x)*], where *d_m,i_(x)* is the degree of “support” given by classifier *C_m_* to the hypothesis that *x* comes from class *i*. The outputs of the classifiers were combined into a so-called decision profile matrix *DP*(*X*):

(13) $$DP(X)=\left\lfloor \begin{array}{c} C_{1}(X)\\ \vdots \\ C_{L}(X) \end{array}\right\rfloor =\left[\begin{array}{ccc} d_{1,1}(X) & \cdots & d_{1,c}(X)\\ \vdots & \ddots & \vdots \\ d_{L,1}(X) & \cdots & d_{L,c}(X) \end{array}\right]$$

The fuzzy template method is closely associated with the training data used. Let *Z = {Z_1_, …, Z_N_*} be the labeled set of training data with N being the total number of samples. The fuzzy template of the class *i* is then defined as the L × c matrix *F_i_*:

(14) $$F_{i}=\left[\begin{array}{ccc} f_{i}(1,1) & \cdots & f_{i}(1,c)\\ \vdots & \ddots & \vdots \\ f_{i}(L,1) & \cdots & f_{i}(L,c) \end{array}\right]$$

whose elements are obtained from:

(15) $$f_{i}(k,s)=\frac{\sum_{j=1}^{N}Ind(Z_{j},i)d_{k,s}(Z_{j})}{\sum_{j=1}^{N}Ind(Z_{j},i)}$$

where *Ind(Z_j_,i)* is an indicator function with value 1 if *Z_j_* comes from class *i* and 0 otherwise. Additionally, k is from 1 to L (number of classifiers); s is from 1 to c (number of classes). Thus, the fuzzy template for class *i* is the average of the decision profiles of the elements of the training set labeled in class *i*. 

The fuzzy template fuses the classifiers and produces the soft class label vector U = [*μ^1^(x), …, μ^c^(x)*] given the new testing data *x* with components:

(16) $$\mu^{i}(x)=S\left(F_{i},DP(x)\right)$$

where *S* is interpreted as similarity. Regarding the two arguments as fuzzy sets, various fuzzy measures of similarity can be used [39]. Here we used

(17) $$\mu^{i}(x)=1-\frac{1}{Lc}{\left(F_{i}-DP(x)\right)}^{2}$$

The final fusion outputs obtained by fuzzy template is the class with maximum soft class label.

## 3. Results

### 3.1. Spectra Characterization

**Figure 3:**
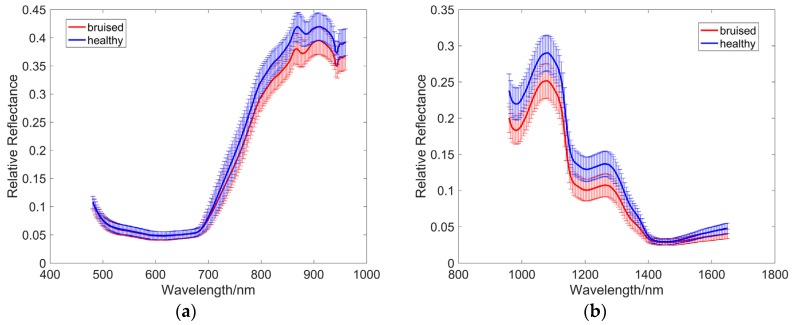
Mean and standard deviation spectra from (**a**) push broom based and (**b**) LCTF based hyperspectral imaging systems.

Hyperspectral reflectance images in the visible region are predominantly affected by the presence of plant pigments [40]. Because both the bruised and healthy portions have very dark skin, the corresponding reflectance intensity was much lower in the spectral region of 480–700 nm (Figure 3). In addition, there was no discrimination between normal and bruised reflectance in the visible region, suggesting that this region was not suitable for bruise detection of blueberries, and that it is challenging to detect bruising of blueberries in RGB images. Therefore, the visible region was removed before further analysis. On the other hand, the larger difference between bruised and unbruised tissues in the spectral region of 700–1650 nm could be used to distinguish between bruised and heathy samples. Three obvious absorption peaks around 980, 1200, and 1460 nm—which are associated with the absorption of water—were observed in the raw reflectance spectra of blueberries.

According to our latest studies on optical properties of healthy and bruised blueberry flesh, the healthy tissues have higher reduced scattering coefficients than bruised tissues over the whole near-infrared region (700–1650 nm) [41], which could explain why the intensity values of the reflectance spectra of healthy tissues were higher than those of bruised tissues. These results are in agreement with the findings in bruise detection of apples [42] and pears [43].

### 3.2. Individual Data Analysis

**Figure 4:**
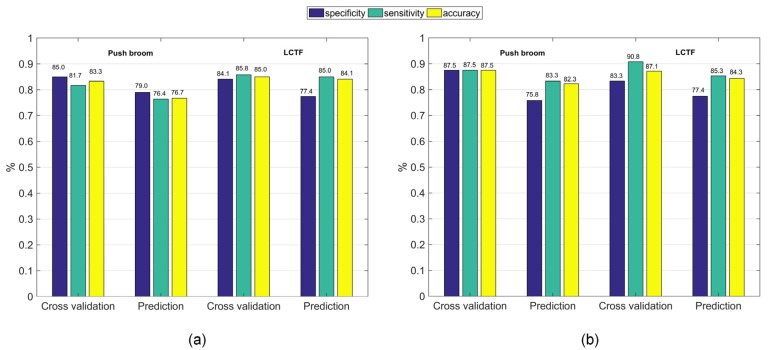
Classification results of (**a**) partial least squares discriminant analysis (PLS-DA) and (**b**) support vector machine (SVM) models for distinguishing between bruised and healthy samples based on the mean reflectance from push broom based and LCTF based HSI.

**Figure 5:**
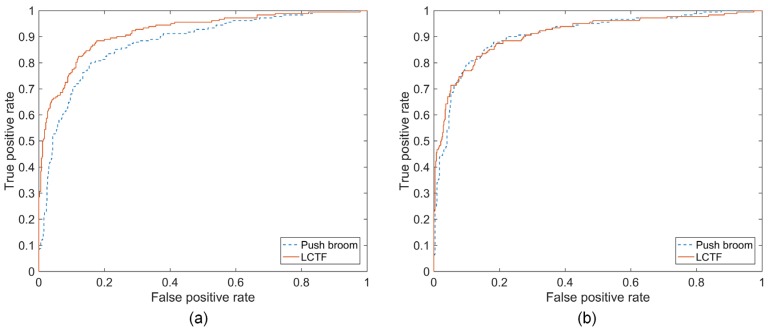
Receiver operating characteristics (ROC) curves of (**a**) PLS-DA and (**b**) SVM classifiers for blueberry bruise detection using mean reflectance from push broom based and LCTF based HSI.

In terms of the classification accuracy obtained by both PLS-DA and SVM analysis in the prediction set (Figure 4), LCTF based HSI outperformed push broom based HSI. At the same time, LCTF yielded higher sensitivity values, indicating that the LCTF was more sensitive for detecting bruised samples. According to the obtained receiver operating characteristics (ROC) curves of PLS-DA and SVM classifiers built with the two HSI systems, the area under curve (AUC) of PLS-DA and SVM classifiers were 0.919 and 0.911, respectively, for the LCTF based hyperspectral data, and 0.877 and 0.909, respectively, for push broom based hyperspectral data (Figure 5). As the AUC of a classifier is equivalent to the probability that the classifier will rank a randomly chosen positive instance higher than a randomly chosen negative instance, the classifier with greater AUC value yields better performance. Hence, the classifiers based on LCTF hyperspectral data achieved better classification results.

The internal tissues after the impact become discolored because of oxidative browning, a result of the release of enzymes during breakdown of cell membranes [44]. The content of chemical groups, such as O–H, C–H, and N–H may change accordingly. The positions of first and second overtones of these functional groups and water are mainly located in the 960–1650 nm region [45]. This could explain why the LCTF based HSI with 960–1650 nm spectral region outperformed push broom based HSI in detecting the bruised samples.

On the other hand, the difference in accuracy between the two HSI systems became much smaller when SVM classifiers with an RBF kernel were applied, suggesting that the push broom based HSI system with an appropriate classification method could have a good performance in blueberry bruising detection. As it is not quite obvious whether there is a more linear relationship or more non-linear relationship between the spectral data and target attributes, the linear and non-linear classifiers’ performances differed accordingly.

For both HSI systems, most misclassified samples were false negatives, in which bruised samples were misclassified as healthy samples (see Supplementary Figure S2). The bruise position of calyx bruise was near the edge of the blueberry instead of in the center of the sample, like stem bruise and equator bruise (Figure 1), making calyx bruise account for a smaller portion of the whole surface during the bruise development compared to stem bruise and equator bruise. Accordingly, the reflectance intensity of the calyx bruise was observed to be higher than the reflectance of stem and equator bruise (see Supplementary Figure S3). As the reflectance of calyx bruise was similar to the healthy reflectance with respect to intensity, calyx bruises were difficult to detect with either push broom based or LCTF based HSI, contributing to false negatives. In addition to blueberries, calyx bruise detection is a challenging task for other fruits, such as apples [23].

### 3.3. Data Fusion

#### 3.3.1. Data Level Fusion

**Figure 6:**
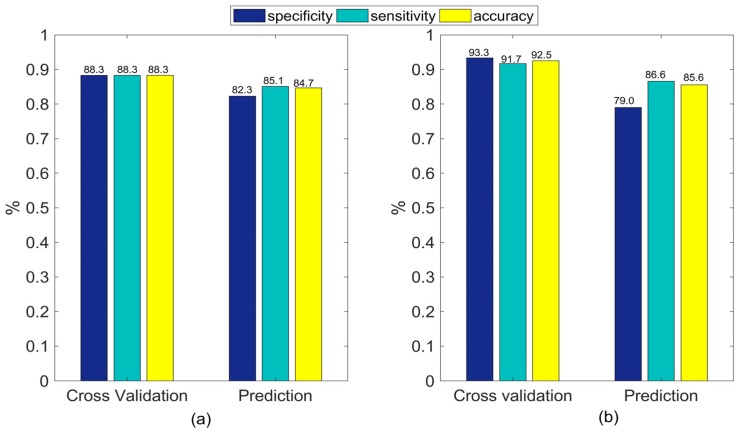
(**a**) PLS-DA and (**b**) SVM classification results of classifiers for distinguishing bruised samples based on the fusion of push based and LCTF based HSI in data level.

In the data level fusion, the individual spectra obtained from the two HSI systems were fused into a single matrix of 240 samples (calibration set) by 241 variables. Moreover, given the fact that the two matrices to be concatenated could have different variance, data were auto-scaled before further analyses. The PLS-DA and SVM models built with data level fusion after autoscaling had increased sensitivity, specificity, and accuracy of cross validation set compared to those obtained by individual sensors (Figure 6). In addition, higher specificity values were obtained after data level fusion in the prediction set, which increased 3.3% and 1.6% for PLS-DA and SVM analysis, respectively. Nevertheless, the enhancement of sensitivity values was only observed for SVM analysis. These improvements after data level fusion might have occurred because the spectral range used for classification was expanded to 700–1650 nm. As mentioned before, both 700–960 nm and 960–1650 nm could provide information to distinguish between bruised and healthy blueberries. Generally, when more information is used, better results are obtained. However, the resulting final matrix after data level fusion may increase the amount of irrelevant or redundant variables brought by the addition of different data sources.

#### 3.3.2. Feature Level Fusion

**Figure 7:**
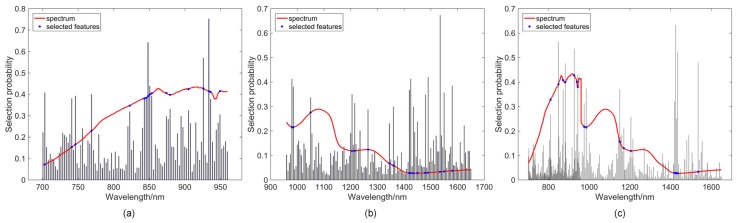
Distribution of features selected by the random frog algorithm from the (**a**) push broom based and (**b**) LCTF based hyperspectral data, and (**c**) their fused data.

The random frog algorithm was carried out on the push broom based and LCTF based hyperspectral data of calibration set separately to select important features carrying the most valuable information related to bruise detection throughout the whole wavelength range of spectra. After calculation of the random frog method, all of the wavelengths were ranked in the descending order of importance based on the selection probability (Figure 7a,b). In order to determine the optimal number of selected features, a series of PLS-DA models with an increasing number of variables were obtained until a predefined maximal number (80) was achieved, followed by computing the classification error of each model using a 10-fold cross validation (see Supplementary Figure S4a,b). The optimal number of variables can then be chosen according to the minimum classification error. After random frog calculation, effective features determined from push broom based HSI system were the following 17 wavelengths: 703.3, 741.1, 746.2, 768.9, 822.6, 843.1, 845.7, 848.3, 850.8, 853.4, 874.0, 879.2, 905.1, 925.9, 933.7, 936.3, 949.3 nm (Figure 7a); and from LCTF based HSI system were the following 17 wavelengths: 980, 985, 1050, 1205, 1215, 1265, 1345, 1360, 1420, 1425, 1435, 1450, 1480, 1490, 1535, 1550, 1580 nm (Figure 7b). Several wavelengths selected by random frog from the push broom based hyperspectral data were around 850 nm, which was also determined for the identification of common defects of peaches [46]. The wavelengths around 740 and 950 nm were also included as the effective features for apple bruise detection [47]. On the other hand, most of the 17 wavelengths from LCTF hyperspectral data were observed to be concentrated at the water absorption bands, such as 980, 1205, and 1450 nm. Water is the main component that varies during the bruising process and most of the optimal wavelengths selected by random frog were related to water absorption, proving the effectiveness of the feature selection method.

After feature selection, a higher accuracy of cross validation was obtained by PLS-DA and SVM classifiers compared with the accuracy using full spectrum (Table 1). In addition, random frog selected LCTF hyperspectral features increased the specificity and accuracy in prediction set, which increased 1.6% and 1.2% for PLS-DA analysis, respectively, and 8.1% and 1.7% for SVM analysis, respectively. In the case of push broom based hyperspectral data, PLS-DA classifiers built with selected features yield comparable accuracy to those based on full wavelength spectra. Although the accuracy obtained by SVM analysis became inferior with a decrease from 82.3% to 80.8%, the specificity increased from 75.8% to 80.6%. As the number of bruised samples was larger than that of healthy samples in prediction set, the accuracy was mainly determined by sensitivity. From this point of view, the overall accuracy would be improved if the numbers of heathy and bruised samples were balanced in the prediction set. All of these results suggested that the selected features could represent most features and characteristics of the whole wavelength to detect blueberry bruising. Most importantly, the classification models based on selected features used only about 16.7% and 12.2% of the variables used by the full-range spectra for the push broom based and LCTF based hyperspectral imaging data, respectively, which reduced the redundant information in hyperspectral images, simplified the classification models, and satisfied the requirements for online bruise inspection.

Meanwhile, the random frog method was applied to the merged matrix to select the features with the highest discriminating power while removing the features that did not contribute to the classification, obtaining 25 effective features (804.7, 809.8, 848.3, 850.8, 871.4, 881.8, 925.9, 938.9, 944.1, 975, 985, 1145, 1150, 1155, 1160, 1170, 1205, 1215, 1420, 1425, 1430, 1435, 1480, 1490, 1535 nm) (see Figure 7c; Supplementary Figure S4c). Afterwards, the features selected separately and jointly were analyzed by PLS-DA and SVM analysis (Table 2). Features selected jointly considered the interaction of the features from the two instruments and reduced the co-linearity of those features, obtaining different effective features and yielding better classification results compared with the features selected separately. Compared to classification results in data level, the classification accuracy in prediction set increased 0.9% and 1.0% for PLS-DA and SVM classifiers, respectively, based on features selected jointly. As feature selection methods captured only the relevant variation in the different data matrices, simplified the classification model, as well as avoided irrelevant or redundant features from the low-level data fusion set, the feature fusion strategy obtained better results than data level fusion in bruise detection.

#### 3.3.3. Decision Level Fusion

The challenge of decision level fusion is to determine the classification models that work best for each data source so that their combination performs better than individual models [14]. The classifiers associated with push broom based and LCTF based HSI may have classification decisions that agree or conflict with each other. In the situation of decision conflict between two classifiers, there must be one correct and one wrong result, and the decision fusion may reduce classification error in such cases using the possibility outputs from the PLS-DA or SVM classifiers.

As classification results in cross validation set were used as the estimated performance on a particular class for individual models, only classification results of prediction set were reported by weighted majority vote, Bayesian network, and fuzzy template (Table 3). Bayesian network, fuzzy template, and weighted majority vote obtained similar classification accuracy (87.1~87.5%) in combining the PLS-DA or SVM classifier results. The fusion results based on SVM classifiers appeared to be more robust than those based on PLS-DA classifiers, when considering the balance between the sensitivity and specificity. It was noted that 84, 85, and 84 out of 101 samples were classified correctly by Bayesian network, fuzzy template, and weighted majority vote, respectively, when the decisions of the two PLS-DA classifiers conflicted with each other. For SVM analysis, 59, 60, and 59 out of 82 samples were identified correctly by the three decision fusion methods, respectively. These results showed that most samples could be classified correctly by decision fusion strategies when the two classifiers had different classification decisions.

## 4. Discussion

The decision level fusion on the basis of feature classification results yielded more promising results than data level and feature level fusion methods. The decision level fusion strategies improved the classification accuracy considerably compared to the classifiers developed using individual instruments, with the accuracy increasing from 84.1% to 87.3% for PLS-DA, and from 84.3% to 87.5% for SVM. All of the decision fusion methods combined the prior performance of push broom based and LCTF based hyperspectral imaging system in blueberry bruise detection with the advantages of utilizing soft evidence output from PLS-DA or SVM. These likelihood values could provide information on the “strength” of its belief that a particular sample belongs to a certain class. Another advantage of this type of fusion is that every individual matrix is processed independently and the results from inefficient techniques do not worsen the overall performance as much as in data or feature levels [14]. Decision level fusion was performed on the classifiers built with relevant features extracted from individual data sources instead of raw signals. Thus, the final identity declaration was obtained from only a few effective features that could represent the relevant and important information of each technique, which combined the advantages of feature level and decision level fusion. As this study demonstrated the feasibility of the fusion of the two HSI systems with complementary spectral ranges for blueberry bruising detection, it is feasible to develop two multispectral systems with the selected wavelengths, and integrate the results from the two systems by using the proposed decision level fusion on a packing line for blueberry bruising detection. Additionally, future work will be conducted to explore the full surface bruise detection without flipping the fruit.

The classification accuracies obtained by decision level fusion in this study were higher than those reported by Hu et al. (2016), who identified mechanical bruising of blueberries using the mean spectrum of each sample extracted from hyperspectral images in the 400–1000 nm spectral range, with classification accuracies of 80.2% and 76.7% for healthy and damaged blueberries, respectively, 12 h after impact. On the other hand, better results were reported in our previous work, which has demonstrated that an LCTF-based hyperspectral reflectance imaging system in the spectral range of 950–1650 nm could be implemented to obtain the spatial distribution of bruises by classifying each pixel of a particular blueberry, to calculate the bruise ratio index for each sample, and to grade the sample accordingly, with the average accuracy of 90% [3]. However, to localize bruising on the fruit using two HSI systems, the hyperspectral images from the two systems should be reconstructed into a single and more complete image. As the field of view and the spatial resolution were different for the two HSI systems, it remains a challenge to fuse the two hyperspectral images at the pixel level and will be addressed in our future studies. Nevertheless, in postharvest quality sorting and grading, it is sufficient to sort blueberries into healthy and bruised classes while unnecessary to localize the bruises for each fruit. Therefore, the mean spectrum of each sample was exacted from each of the HSI systems separately and then fused at the data, feature, and decision level in this study. 

Although this study was only limited to the fusion of the spectral data from two imaging technologies, the application of the methodology (in particular, decision level fusion) presented here could be expanded to other studies, as it just integrated different classifier results and did not consider the structure of collected data from different sensors. Given that more non-destructive methods, such as thermal imaging, MRI, and X-ray imaging [48], have been successfully used for bruise detection of fruits, it would be an interesting task to explore blueberry bruise detection by fusing these different technologies using the proposed fusion methods. 

## 5. Conclusions

The data fusion strategies at all three levels achieved better classification results than using push broom based and LCTF based HSI individually. The decision level fusion based on classification results with selected features yielded the best final classification results, demonstrating that the information obtained from the two imaging spectroscopic techniques has a synergistic effect. Based on the HSI-selected wavelengths and proposed methodology, multispectral imaging systems could be developed and fused to detect blueberry bruising on the packing line. Although this paper focused on blueberry bruise inspection, it should be noted that the data fusion strategies presented in this study are generally suitable to other food applications.

## Figures and Tables

**Table 1:** Classification results of PLS-DA and SVM models distinguishing between bruised and healthy samples based on the effective features selected from push broom based and LCTF based HSI separately.

Classifier	Data	No. of Features	Cross Validation Set			Prediction Set		
			Specificity	Sensitivity	Accuracy	Specificity	Sensitivity	Accuracy
PLS-DA	Push broom	17	89.2%	86.7%	87.9%	79.0%	76.1%	76.5%
	LCTF	17	90.0%	90.0%	90.0%	79.0%	86.3%	85.3%
SVM	Push broom	17	89.2%	87.5%	88.3%	80.6%	80.8%	80.8%
	LCTF	17	93.3%	88.3%	90.8%	85.5%	86.1%	86.0%

**Table 2:** Classification results of PLS-DA and SVM models distinguishing between bruised and healthy samples based on the fusion of push broom based and LCTF based HSI jointly at feature level.

Classifier	Schemes	No. of Features	Cross Validation Set			Prediction Set		
			Specificity	Sensitivity	Accuracy	Specificity	Sensitivity	Accuracy
PLS-DA	Features selected jointly	25	93.3%	91.2%	92.5%	82.3%	86.1%	85.6%
	Features selected separately	34	90.8%	90.0%	90.4%	82.3%	85.1%	84.7%
SVM	Features selected jointly	25	92.5%	91.7%	92.1%	83.9%	87.1%	86.6%
	Features selected separately	34	90.8%	91.7%	91.3%	80.6%	85.6%	84.9%

**Table 3:** Classification results of prediction set obtained by fusing the feature level classification using decision fusion methods.

Classifier	Decision Fusion Methods	$N_{c1=c2}^{\mathrm{correct}}$/N_c1 = c2_	$N_{c1\neq c2}^{\mathrm{correct}}$/N_c1≠c2_	Specificity	Sensitivity	Accuracy
PLS-DA	Bayesian network	320/353	84/111	82.2%	87.8%	87.1%
	Fuzzy template	320/353	85/111	82.2%	88.1%	87.3%
	Weighted majority vote	320/353	84/111	82.2%	87.8%	87.1%
SVM	Bayesian network	346/382	59/82	85.5%	87.6%	87.3%
	Fuzzy template	346/382	60/82	83.9%	88.1%	87.5%
	Weighted majority vote	346/382	59/82	85.5%	87.6%	87.3%

$N_{c1=c2}^{\mathrm{correct}}$, number of samples that were correctly predicted when the two classifiers make the same decision. N_c1 = c2_, number of samples that the two classifiers make the same decision. $N_{c1\neq c2}^{\mathrm{correct}}$, number of samples that were correctly predicted when the two classifiers make the conflicted decision. N_c1≠c2_, number of samples that the two classifiers make the conflicted decision.

## Supplementary Materials

The following are available online at http://www.mdpi.com/1424-8220/18/12/4463/s1: Figure S1: PLS-DA probability output. Figure S2: False negative rate of stem, equator, and calyx bruise groups obtained by (a) PLS-DA and (b) SVM analysis based on the mean reflectance from push broom based and LCTF based HSI. Figure S3: Mean reflectance of stem, equator, and calyx bruise collected by (a) push broom based and (b) LCTF based HSI. Figure S4: The classification error of 10-fold cross validation calculated by the PLS-DA for the features generated by random frog from (a) push broom based and (b) LCTF based hyperspectral data, and (c) their fused data. Table S1: Number of blueberries used in HSI experiment.
