# Supplementary material for: Data Fusion of Two Hyperspectral Imaging Systems with Complementary Spectral Sensing Ranges for Blueberry Bruising Detection
Source: Sensors (Basel). 2018 Dec 17;18(12):4463. doi: 10.3390/s18124463 (PMC6308671; doi:10.3390/s18124463)
Supplement: Supplementary file 1 [file sensors-18-04463-s001.pdf]

Data Fusion of Two Hyperspectral Imaging Systems with Complementary Spectral Sensing Ranges for Blueberry Bruising Detection

Supplementary Materials

Shuxiang Fan[1, 2], Changying Li[2*], Wenqian Huang[1], Liping Chen[1]

[1] Beijing Research Center of Intelligent Equipment for Agriculture, Beijing, China

[2] Bio-Sensing and Instrumentation Laboratory, College of Engineering, 712F Boyd Graduate Studies, University of Georgia, Athens, Georgia, 30602, United States of America

[*]Corresponding author: 712F Boyd Graduate Studies, 200 D. W. Brooks Drive, University of Georgia, Athens, Georgia, 30602, United States of America.

Phone: (706) 542-4696; Fax: (706) 542-2475; Email: cyli@uga.edu;

Website: http://sensinglab.engr.uga.edu/

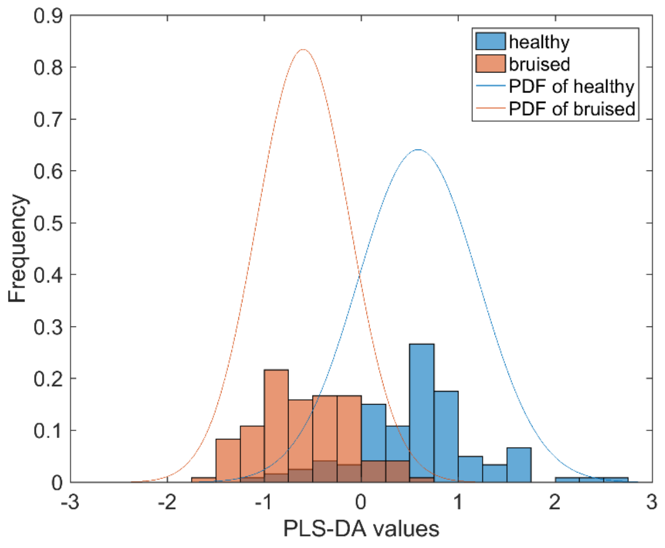

Figure S1. PLS-DA probability output.

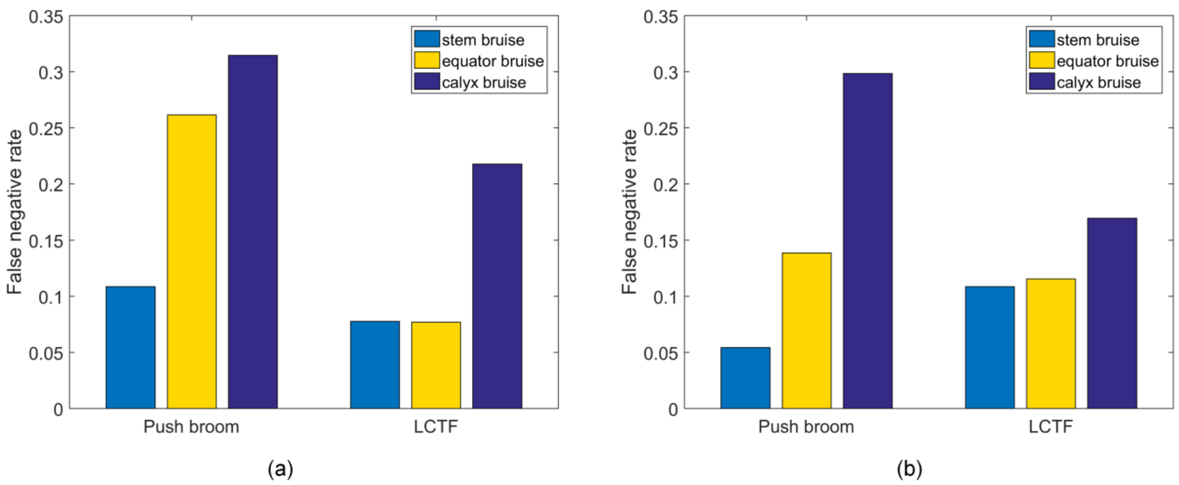

Figure S2. False negative rate of stem, equator, and calyx bruise groups obtained by (a) PLS-DA and (b) SVM analysis based on the mean reflectance from push broom based and LCTF based HSI.

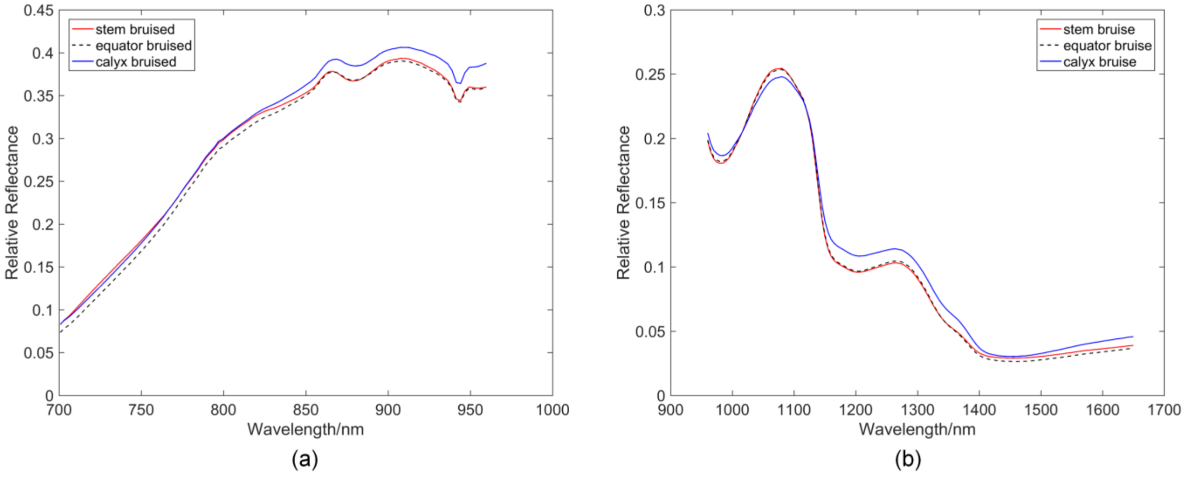

Figure S3. Mean reflectance of stem, equator, and calyx bruise collected by (a) push broom based and (b) LCTF based HSI.

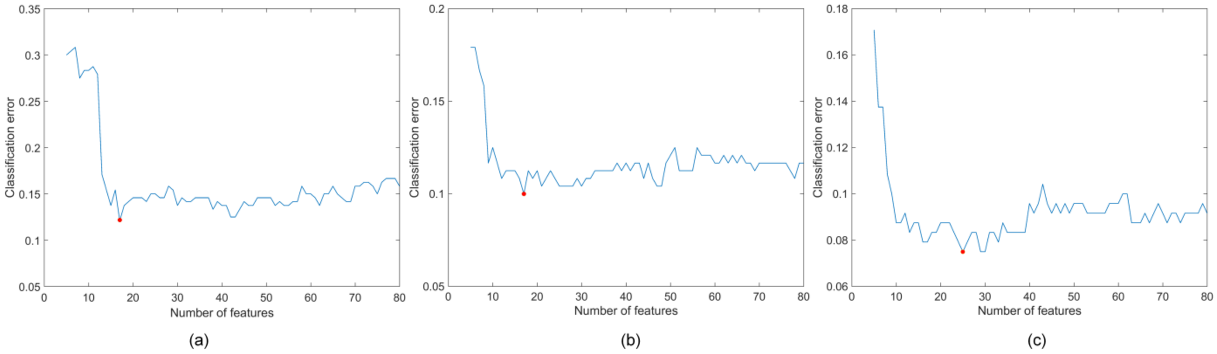

Figure S4. The classification error of 10-fold cross validation calculated by the PLS-DA for the features generated by random frog from (a) push broom based and (b) LCTF based hyperspectral data, and (c) their fused data.

Table S1. Number of blueberries used in HSI experiment.

Variety	Calibration set		Prediction set	
	Control	Bruised	Control	Bruised
Bluecrop	60	60	40	160
Jersey	60	60	26	238
